# Inheritance of the 8.1 ancestral haplotype in recurrent pregnancy loss

**DOI:** 10.1093/emph/eov031

**Published:** 2015-12-16

**Authors:** Astrid M. Kolte, Henriette S. Nielsen, Rudi Steffensen, Bernard Crespi, Ole B. Christiansen

**Affiliations:** ^1^Recurrent Pregnancy Loss Unit, Fertility Clinic 4071, University Hospital Copenhagen Rigshospitalet, Blegdamsvej 9, Copenhagen Ø 2100, Denmark;; ^2^Department of Clinical Immunology, Aalborg University Hospital, North, Urbansgade 32, Aalborg 9000, Denmark;; ^3^Human Evolutionary Studies Program and Department of Biological Sciences, Simon Fraser University, 8888 University Drive, Burnaby, BC V5A 1S6, Canada and; ^4^Department of Gynecology and Obstetrics, Aalborg University Hospital North, Reberbansgade 15, Aalborg 9000, Denmark

**Keywords:** recurrent pregnancy loss, gestational drive, selfish gene theory, cohort study, mother–offspring conflict

## Abstract

**Background and objectives:** The 8.1 ancestral haplotype (AH) (HLA-A1, C7, B8, C4AQ0, C4B1, DR3, DQ2) is a remarkably long and conserved haplotype in the human major histocompatibility complex. It has been associated with both beneficial and detrimental effects, consistent with antagonistic pleiotropy. It has also been proposed that the survival of long, conserved haplotypes may be due to gestational drive, i.e. selective miscarriage of fetuses who have not inherited the haplotype from a heterozygous mother. Recurrent pregnancy loss (RPL) is defined as three or more consecutive pregnancy losses. The objective was to test the gestational drive theory for the 8.1AH in women with RPL and their live born children.

**Methodology:** We investigated the inheritance of the 8.1AH from 82 heterozygous RPL women to 110 live born children. All participants were genotyped for HLA-A, -B and -DRB1 in DNA from EDTA-treated blood or buccal swaps. Inheritance was compared with a Mendelian inheritance of 50% using a two-sided exact binomial test.

**Results:** We found that 55% of the live born children had inherited the 8.1AH, which was not significantly higher than the expected 50% (*P* = 0.29). Interestingly, we found a non-significant trend toward a higher inheritance of the 8.1AH in girls, 63%, *P* = 0.11 as opposed to boys, 50%, *P* = 1.00.

**Conclusions and implications:** We did not find that the 8.1AH was significantly more often inherited by live born children of 8.1AH heterozygous RPL women. However our data suggest that there may be a sex-specific effect which would be interesting to explore further, both in RPL and in a background population.

## Abstract (precis)

A segment of DNA called the 8.1 ancestral haplotype is hypothesized to cause fetal loss due to a ‘selfish gene’ effect. The hypothesis was not supported, although the haplotype tended to be inherited more often than expected among girls (p=0.11) in a study of 110 mother-child pairs. Further studies are warranted.

## INTRODUCTION

A haplotype is a contiguous set of alleles which tends to be inherited together without recombination. The 8.1 ancestral haplotype (AH) (HLA-A1, C7, B8, C4AQ0, C4B1, DRB1*0301, DQ2) is carried by approximately 10% of Northern Europeans. The 8.1AH is remarkably long, spanning 2.9 MB and has >99.9% conservation [1]. It has been suggested that the high prevalence of the haplotype in Caucasian populations is due to increased resistance to infections, as the 8.1AH has been associated with a longer time to permanent infections with bacteria in the lungs of patients with mucoviscidosis (cystic fibrosis) [2, 3] and a protective role against multi-organ failure (septic shock) in patients with blood infection (sepsis) due to bacterial lung infections (pneumonia) [4]. However, it also leads to an increased susceptibility to HIV and a higher frequency of progression to AIDS [5] and risk of field fever (leptospirosis) [6]. Furthermore, the 8.1AH is associated with a number of autoimmune diseases, such as toxic diffuse goiter (Grave’s disease) and systemic lupus [7, 8]. The 8.1AH’s effects on health and disease are in some cases sex-dependent. Some effects of the 8.1AH appear sex specific: female, but not male, carriers of the 8.1AH have a higher risk of cancer in the distal gut [9] and early onset myasthenia gravis, an autoimmune neuromuscular disease which leads to muscle weakness and fatigue [10]. Likewise, patients with sporadic inclusion body myositis (an inflammatory muscle disease with progressive muscle wasting and weakening) and concurrent Sjögren’s syndrome (an autoimmune disease where the exocrine glands are destroyed, leading to dry eyes and mouth) were found to be predominantly female carriers of the 8.1AH [11]. Conversely, the 8.1AH seems to be associated with longevity, but only in men [12]. Of interest in reproductive immunology, fetal carriage of the 8.1AH has been associated with higher birth weight [13].

These diverse effects may be consistent with the theory of antagonistic pleiotropy; that a gene or haplotype has both beneficial and deleterious effects in the same individual [14]. If the positive effects on average outweigh the negative effects, the haplotype persists. There is evidence that the 8.1AH stems from a single ancestor rather than recombination [8, 15, 16].

### Gestational drive and the human MHC

**Figure 1.**
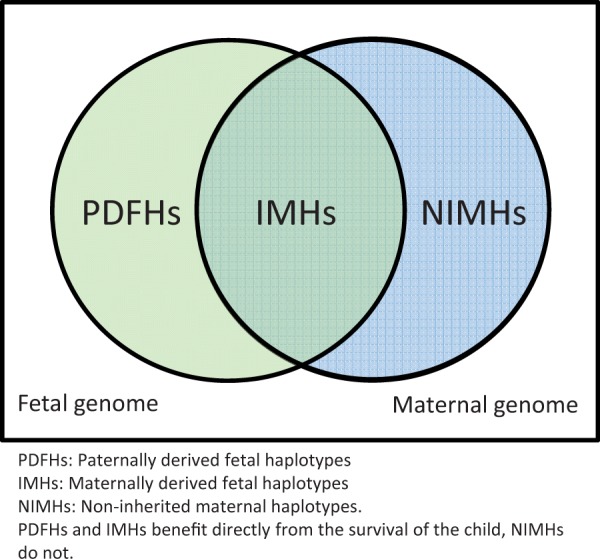
Mother–offspring conflict in viviparous pregnancy. PDFHs and IMHs benefit directly from the survival of the child, NIMHs do not. See also Ref. [17]

In all viviparous pregnancies, there are three interacting genetic compartments. These are the inherited maternal haplotypes (IMHs), non-inherited maternal haplotypes (NIMHs) and paternally derived fetal haplotypes (PDFHs) [17] (Fig. 1). These genetic compartments may not always have the same optimal outcome of the pregnancy. In a given pregnancy, the IMHs and PDFHs benefit directly from the survival of the fetus, as they are present in the fetus, in contrast to the NIMHs. Gestational drive is described as the ability of maternal genes or haplotypes to favor those offspring who carry their replicas [18] and it has been proposed that NIMHs may be responsible for selective miscarriage of the present fetus in order to increase the chances of their own propagation (via the next pregnancy). The abortifacient maternal haplotype would benefit if reproductive compensation is present [19]. According to Haig [18, 20], this system of ‘spiteful abortion’ would be more likely to occur in the large, conserved haplotypes present in the MHC. One of these is the 8.1AH.

Women with recurrent pregnancy loss (RPL) would be a suitable sub-population within which to search for evidence of gestational drive [17]. RPL is defined as three or more consecutive early pregnancy losses and is a heterogeneous condition with an unknown etiology for the majority of patients after standard evaluation [21]. The majority of women with RPL has only experienced pregnancy losses before 22 weeks’ gestation (primary RPL), but 40% of women with RPL have a live- or stillborn child after 22 weeks’ gestation before the series of early pregnancy losses, ‘secondary RPL’ [22]. Half of the pregnancy losses are aneuploid [23], the frequency of which decreases with increasing number of pregnancy losses in the history [24] and among younger patients [23]. These findings suggest that non-chromosomal causes dominate in younger patients and patients with multiple (> 4) pregnancy losses.

Polymorphisms in both classical and non-classical HLA loci have been reported to play a role in RPL pathogenesis [25–27]. We have found that women with secondary RPL or with ≥4 pregnancy losses were significantly more often carriers of the HLA-DRB1*03 allele compared with controls [26]. HLA-DRB1*0301 is part of the 8.1AH but in this earlier study, typing of HLA class I alleles was not performed, nor were live born children or miscarried fetuses HLA typed. In the present study, the objective was to investigate whether there was a preferential inheritance of the 8.1AH from heterozygous women to their live born children. We investigated this hypothesis within a 30-year national RPL cohort.

## METHODOLOGY

RPL was defined as three or more consecutive pregnancy losses, including both non-visualized pregnancy losses (biochemical pregnancy losses and pregnancies of unknown location combined) and confirmed intrauterine miscarriages [28]. The inclusion criteria for the study were regular menstrual cycles (21–34 days), normal uterine anatomy, at least one of the pregnancy losses had to be a verified intrauterine miscarriage, normal karyotype (also the partner), negative test for lupus anticoagulant and IgG anticardiolipin antibody <45 GPL-U. Furthermore, patients had to be younger than 40 years of age at referral, of Caucasian descent and have at least one live born child at the time of the study. Data about subsequent pregnancy outcome were collected by questionnaires returned by the patients after they had given birth and/or from the Danish national birth register.

As part of standard clinical evaluation, all patients in the Danish RPL Unit are genotyped for HLA-DRB1.

In a previous study of women with unexplained RPL after the birth of a live- or still born child (secondary RPL), 358 women and 203 of their firstborn children were genotyped for HLA-A, -B and -DRB1 [27]. Of these 203 mother–child pairs, 35 women were heterozygous for the 8.1AH. These 35 mother–child dyads were included in the present study and formed the basis for the power calculation.

We went through all files of patients seen from January 1990 to April 2015 in the Danish RPL Unit (approximately 2000 patients) and identified, in addition to the 35 women mentioned above, women who were homo- or heterozygous for HLA-DRB1*03 and had had at least one live born child either before or after their series of pregnancy losses (*n* = 185). Patients who were heterozygous or homozygous for HLA-DRB1*03 were genotyped for HLA-A and -B. Of these, 55 were heterozygous for the 8.1AH. These women were invited to participate in the study and 47 accepted. They had a total of 80 children, from whom we were able to collect samples from 75. Combined with the previous cohort, we included 110 mother–child pairs. We gathered information on birth weight from 90 of the live born children (82%). In all dyads, identity by descent of the 8.1AH was unequivocally ascertained without the need for paternal karyotyping, which was not performed. One child was homozygous for the 8.1AH, but otherwise, none of the included children had inherited the 8.1AH from their fathers.

Written informed consent was obtained. The study was approved by the Regional Ethics Committee for the Capital Region of Denmark, with approval number H-2-2011-055 and by the Danish Data Protection Agency, file number 2007-58-0015.

### Laboratory methods

EDTA-treated peripheral blood (women and adult children) or buccal swaps (children younger than 18 years of age) were collected. DNA from blood was extracted either using a salting-out method as previously described [29] or using the Maxwell 16 Blood DNA kit on the Maxwell 16 Instrument. DNA from buccal swabs was extracted using Maxwell 16 Buccal Swab LEV DNA Purification Kit on the Maxwell 16 Instrument (Promega, Madison, WI, USA).

HLA-A, -B and -DRB1 genotypes were determined by the Luminex xMAP system LABType SSO, a reverse SSO DNA typing system (One Lambda Inc., Canoga Park, CA, USA) according to the manufacturer’s instructions.

### Power calculation

According to the Mendelian laws, we would expect the inheritance of the 8.1AH to be 50%, i.e. *P*_ex_*_p_* = 0.5. The power calculation was performed as a two-sided one-sample inference to a known proportion. In our earlier study we found that of the 35 women with secondary RPL, 22 had bequeathed the 8.1AH to their live born child (63%). Therefore, we set the *P*_obs _= 0.65. With a power of 0.9 and a type I error of 0.05, 113 mother–child pairs were required.

### Statistics

To test whether the 8.1AH was significantly more often bequeathed from RPL women to their children than expected, we used the two-sided exact binomial test. The non-parametric median test was used to assess differences in birth weight and the χ^2^ test was used to test sex-ratio. All statistical analyses were performed in the Statistical Package for Social Sciences (IBM SPSS, Armonk, NY, USA).

## RESULTS

In our cohort of 110 mother–child pairs, we found that 61 (55%) of the live born children had inherited the 8.1AH, which was not significantly higher than the expected 50%, *P* = 0.25. According to the power calculation, we should have included a total of 113 mother-–child pairs. If we assume that all of the remaining three children had inherited the 8.1AH, the proportion would be 57%; *P* = 0.19.

Among the children, there was a trend toward more boys than girls (62 vs 48, *P* = 0.34). There was no trend toward a higher inheritance among boys, 50%, *P* = 1.00. Among girls there was a non-significant trend toward higher inheritance, 63%, *P* = 0.11. In a one-sided test this would be almost significant, *P* = 0.055.

We also analyzed the data for children born by women with primary RPL and secondary RPL separately. We found no statistically significant differences in inheritance, *P* = 0.68 and *P* = 0.39, respectively. Among children born before the series of pregnancy losses, 19 (50%) of the boys and 18 (60%) of the girls had inherited the 8.1AH. In the group of children born after the pregnancy losses, the numbers were not significantly different: 12 (50%) of the boys and 12 (67%) of the girls had inherited the 8.1AH from their heterozygous mother; *P* = 1.00 for boys and *P* = 0.64 for girls.

Furthermore, we ascertained whether the birth weight of children in this cohort was related to their carriage of the 8.1AH. As birth weight is dependent on sex, we stratified the information according to this. However, we found no significant differences in birth weight according to inheritance of the 8.1AH.

The median number of pregnancy losses at referral was 3 (interquartile range 3; 5) and we investigated the inheritance of the 8.1AH stratified for the mothers’ number of pregnancy losses prior to referral. We saw no significant differences, nor any trends.

The results are summarized in Table 1.

**Table 1.** Inheritance of the 8.1 AH from RPL women to their live born children

	Inherited *N* (%)	Did not inherit *N* (%)	*P* value
All live born children (*N* = 110)	61 (55%)	49 (45%)	0.29^a^
Type of RPL			
Secondary RPL (*n* = 87)	48 (55%)	39 (45%)	0.39^a^
Primary RPL (*n* = 23)	13 (57%)	10 (43%)	0.68^a^
Median birth weight (range)^b^			
Boys (*n* = 53)	3320 (1992; 4270)	3481 (1000; 5300)	0.90^c^
Girls (*n* = 37)	3300 (1240; 4800)	3100 (2440; 3675)	0.37^c^
Sex of live born			
Boys (*n* = 62)	31 (50%)	31 (50%)	1.0^a^
Girls (*n* = 48)	30 (63%)	18 (37%)	0.11^a^
Pregnancy losses before referral (number of women)			
3 (n = 43)	25 (58%)	18 (39%)	0.36^a^
4 (n = 25)	13 (52%)	12 (48%)	1^a^
5 or more (n = 42)	23 (55%)	19 (45%)	0.64^a^

^a^Exact binomial test.

^b^We did not have information on birth weight for all live born children.

^c^Non-parametric median test.

## DISCUSSION

We tested the gestational drive hypothesis for the 8.1AH in a cohort of women with unexplained RPL and their live born children. We did not find a significantly higher degree of inheritance of the 8.1AH among children born by heterozygous women with RPL. It is evident that statistical power is limited, especially in the subgroup analyses.

The study is based on a well-known hypothesis of gestational drive [20]. The studied cohort consists of clinically well-characterized patients with no known risk factors for their pregnancy losses and their live born children. We have included both women with primary and secondary RPL. Women with secondary RPL may be somewhat different from women with primary RPL in their HLA background and especially HLA-DRB1*03 frequency [26]. It would be interesting to include a sufficient number of mother–child pairs to evaluate the groups separately. On the other hand, one could argue that if the theory of gestational drive holds true, there should be no difference between women with secondary and primary RPL as the first fetus is as likely to inherit the 8.1AH from the heterozygous mother as the fourth fetus.

In the present study, we have not investigated the inheritance of the 8.1AH from the children’s fathers. However, in future studies of gestational drive and parent–offspring conflict in general, it would be interesting to include all genetic shareholders.

Regrettably, we do not have access to pregnancy loss tissue from the vast majority of our patients, neither before nor after referral. As these women have more failed than successful pregnancies, investigating the miscarried pregnancies for carriage of the 8.1AH, and in addition chromosomal aberrations, would have strengthened the study. This would also have enabled us to include women with exclusively or predominantly euploid pregnancy losses.

We found a trend toward higher inheritance of the 8.1AH among live born girls, *P* = 0.11. As we tested a hypothesis with a specified directionality (i.e. the 8.1AH being inherited more often than expected by Mendelian inheritance), it could be argued that a one-sided test would be appropriate. This would have yielded a *P*-value of 0.055 in the subgroup analysis of the girls. However, we could not *a priori* exclude the possibility that the 8.1AH was bequeathed less often than expected, and therefore we chose the two-sided binomial test.

We have previously shown that firstborn children born by women with secondary RPL are significantly more often boys. Children born after the series of pregnancy losses are significantly more often girls than boys compared with the expected 1.06 sex ratio [30]. Furthermore, women with secondary RPL have a higher chance of live birth in the first pregnancy after referral if the firstborn is a girl [27]. Immunity to male-specific minor histocompatibility (HY) antigens and maternal carriage of the HY restricting HLA class II alleles HLA-DRB1*15, -DQB1*05:01/02 play a significant role in secondary RPL following the birth of a boy, but not a girl [22, 27]. Therefore, *a priori*, we would not necessarily expect that the 8.1AH is associated with secondary RPL following the birth of a boy. Both among RPL women and on a population basis, we have also found that a firstborn boy leads to a lower birth weight of later born sibs, especially if the later born child is a boy [31, 32]. Therefore, gestational drive in RPL may be more prominent in secondary RPL after a firstborn girl as HY immunity does not seem to play a large role in this subset of patients. Our finding of a trend toward increased inheritance of the 8.1AH among live born girls (*P* = 0.11) is also interesting taking into consideration that others have reported sex-specific effects of the 8.1AH [9–12]. The gestational drive hypothesis does not specify sex-specific effects, although sibling rivalry may be more intense within sexes rather than between them (D. Haig, personal communication).

## CONCLUSIONS AND IMPLICATIONS

To our knowledge, this is the first attempt to identify the highly conserved 8.1AH as a ‘selfish gene’ [33] in a human cohort. The lack of a significantly higher inheritance of the 8.1AH by live born children of women with RPL does not prove the hypothesis wrong. As outlined above, there are several other plausible explanations. We found a near-significant trend toward an increased inheritance in the order of 13% among live born girls. For the etiology of RPL, the impact of gestational drive by the 8.1AH seems negligible from a clinical point of view. However, even seemingly weak effects could theoretically explain the prevalence of the 8.1AH in Caucasian populations. As such this type of study is important for empirical testing of evolutionary hypotheses of mother–offspring conflict in human reproduction.

## FUNDING

This work was supported by the Faculty of Health Sciences of the University of Copenhagen by a PhD fellowship to A.M.K.; grants from the Obel Foundation and the AP Møller Foundation supported the laboratory work.

**Conflict of interest**: None declared.
